# Perspectives on Home-based Healthcare as an Alternative to Hospital Admission After Emergency Treatment

**DOI:** 10.5811/westjem.2017.3.32348

**Published:** 2017-05-15

**Authors:** Amy Stuck, Christopher Crowley, Tracy Martinez, Alan Wittgrove, Jesse J. Brennan, Theodore C. Chan, Edward M. Castillo

**Affiliations:** * West Health Institute, Health Services, Successful Aging, La Jolla, California; † University of California, San Diego, Department of Emergency Medicine, San Diego, California

## Abstract

**Introduction:**

The study objective was to explore emergency physicians’ (EP) awareness, willingness, and prior experience regarding transitioning patients to home-based healthcare following emergency department (ED) evaluation and treatment; and to explore patient selection criteria, processes, and services that would facilitate use of home-based healthcare as an alternative to hospitalization.

**Methods:**

We provided a five-question survey to 52 EPs, gauging previous experience referring patients to home-based healthcare, patient selection, and motivators and challenges when considering home-based options as an alternative to admission. In addition, we conducted three focus groups and four interviews.

**Results:**

Of participating EPs, 92% completed the survey, 38% reported ordering home-based healthcare from the ED as an alternative to admission, 90% ranked cellulitis among the top three medical conditions for home-based healthcare, 90% ranked “reduce unnecessary hospitalizations and observation stays” among their top three perceived motivators for using home-based care, and 77% ranked “no existing process in place to refer to home-based care” among their top three perceived barriers. Focus group and interview themes included the need for alternatives to admission; the longer-term benefits of home-based healthcare; the need for streamlined transition processes; and the need for highly qualified home-care staff capable of responding the same day or within 24 hours.

**Conclusion:**

The study found that EPs are receptive to referring patients for home-based healthcare following ED treatment and believe people with certain diagnoses are likely to benefit, with the dominant barrier being the absence of an efficient referral process.

## INTRODUCTION

Clinicians, researchers, and government agencies recognize the need to move toward value-based healthcare delivery that supports population health, better matches the needs of an aging population, and reduces the emerging burden of healthcare costs for the U.S.^1^ Since the release of the Medicare Access and CHIP Reauthorization Act (MACRA) final rule in 2016, providers are increasingly incentivized by Medicare to assume more risk, not only to save money but to provide care that is more person-centered, higher quality, and more affordable. Seniors are also shifting from traditional fee-for-service Medicare to Medicare Advantage plans, the latter of which often provide access to additional benefits such as medications and other services.^2^

Of the $3 trillion spent per year on healthcare in the U.S., $2 trillion is spent on chronic disease, with inpatient medical care accounting for 31% of nationwide health spending ($1 trillion per year).^3^ Emergency departments (ED) are the primary entry point for hospital admissions. In effect, emergency physicians (EP) act as gatekeepers in the decision to admit patients to hospitals.^4^ Specifically, over half of all hospital admissions and nearly 70% of senior admissions occur through the ED.^5^,^6^

Currently, EPs are generally afforded only three disposition options for patients presenting to the ED: (1) discharge, (2) observe, or (3) admit. While admission may represent the most appropriate option for many acute conditions, there also exist risks of “default” admissions, where there are simply no other options for a needed course of therapy or monitoring following ED evaluation and management. In some instances, admission may not be the best match for ongoing management of multiple chronic conditions, especially in the frail elderly where there is an elevated risk of in-hospital nosocomial infections, delirium, and falls.^7^

There is an emerging need to provide EPs with home-based healthcare alternatives to hospital admission, particularly for the elderly. The first objective of the study was to investigate the level of EP’s awareness about their ability to refer patients to home-based healthcare, and the EPs’ experience, if any, in invoking a home-health referral. The next objective was to uncover EP motivations for considering the use of home health for ED patients versus hospital admission, the type of patients EPs select, and time requirements for an initial home-health visit. The final objective was to gain knowledge, through conducting focus groups and interviews that paired EPs with key ED clinicians and decision makers about the incentives or barriers for using home care as an alternative to hospital admission. An analysis of the potential cost basis for home-based care as an alternative to hospitalization is presented elsewhere.^8^

## METHODS

### Study Design and Participants

We used an email survey, a focus group, and interview methodologies to explore knowledge of and willingness to use appropriate and safe home care as an alternative to hospital admission. The research setting included two EDs in an academic healthcare system. One hospital was an urban academic teaching hospital (Level I trauma center) with an annual census of 48,000 visits. The second was a suburban community hospital with an annual census of 27,000 visits. An Emergency Medicine Physician Survey was electronically distributed to all 52 attending physicians practicing in the health system’s EDs between August and September 2014. A total of 48 (92.3%) physicians responded to the survey.

**Population Health Research Capsule**

What do we already know about this issue?

We know that most hospital admissions originate in the ED. We also know that repeated hospitalizations for older adults elevate the risk of nosocomial infections, delirium, and falls.

What was the research question?

What is the emergency physician’s (EP) perspective on disposition to home-based care following evaluation and management in the ED?

What was the major finding of the study?

EPs are receptive to referring patients for home-based care, but few have access to processes for doing so.

How does this improve population health?

It shows the potential to reduce hospital admissions from the ED, which could allow more patients—especially seniors—to be cared for at lower cost in the comfort and safety of their own homes.

For the focus groups and interviews, study participants included three EPs, with the addition of ED leadership overseeing operations and quality, registered nurses (RNs) and social workers (SWs) employed in the ED. The focus of this research was confined to patients being evaluated in the ED.

### Selection of Focus Group Participants

The answers on the physician survey were, in part, used to develop the focus group interview guides. We selected physician participants if, on the survey, they indicated previous experience with referrals to home care as an alternative to admission. Of the 48 EPs who responded, 18 (37.5%) indicated prior experience with referring patients directly from the ED. All focus group participants were selected on their availability to participate during the time frame of December 2014 through February 2015. The study was approved by the institution’s Human Research Protection Program.

### Study Procedures and Data Analysis

#### Survey

We developed survey questions following qualitative data gathered from more than 40 telephone interviews with emergency physicians and nurses, hospital and home-health leaders, case managers, primary care physicians, and health plan administrators from across the U.S. Survey answer choices such as barriers to home-health referrals and conditions amenable to home health were gleaned from these preliminary interviews. We designed the survey questions to gauge EPs’ awareness of and prior experience with referring to home-based care as an alternative to hospital-based care. Physicians were invited to complete the survey via e-mail. The survey consisted of five questions. The first question determined whether EPs had prior experience discharging patients to home-based healthcare directly from the ED. If they answered yes, the next question asked their reasons for this decision. The following two questions ascertained what motivations EPs had for considering a home-based referral, as well as the types of clinical diagnoses they would consider eligible. Finally, the survey inquired about the necessary response time the home-based care staff would need to provide for EPs to consider care at home a safe option.

#### Focus Groups

The primary research team conducted three focus groups and four interviews, each approximately one hour in duration, using a discussion guide. Focus groups and interviews were audiotaped and then transcribed verbatim. The research team conducted thematic analysis using a *horizontal* approach.^9^ Four members of the research team independently read all focus group and interview transcripts to identify patterns of recurrent concepts. The team then sorted and coded the transcriptions, categorizing comments into major and minor themes based on frequency of similar comments from participants. Major themes and concepts were finalized by consensus.

## RESULTS

### Emergency Physician Survey

**Figure 1:**
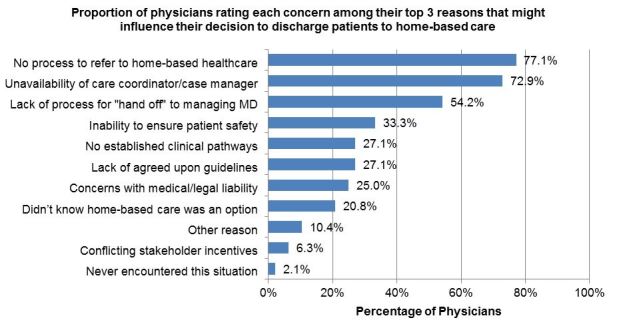
Physician rating of top three reasons influencing decision to choose home-based care. Other reasons listed: Feasibility to get PICC line and appropriate antibiotics delivered (1); Just not something that most of us are familiar with (1); Lack of emergency physician support to execute plan (1); Takes too much time to arrange in a busy ED (1); Unclear if patient’s funding covers services (1).

Of the 48 respondents, 30 (62.5%) EPs indicated no prior experience ordering home-based care from the ED. When respondents were asked to indicate the three most important reasons or concerns that might influence their decision to discharge patients to home-based care (10 options were provided), the most common concern reported was “no existing process in place to refer to home-based care,” with 37 (77.1%) physicians rating this in their top three responses. The second most common response was “unavailability of a care coordinator/case manager*”*; 35 (72.9%) physicians rated this reason among their top three. See Figure 1.

**Figure 2:**
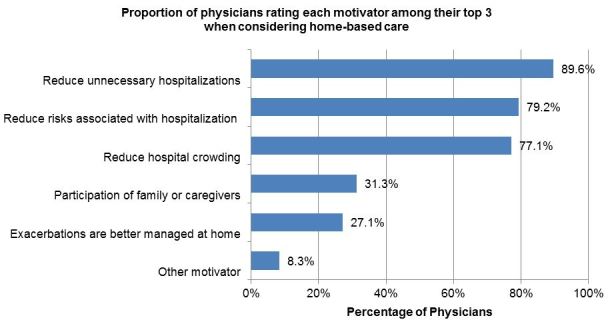
Physician rating of top three motivators when considering home-based care. Other motivators listed: Lower cost for equivalent care (1); Patient preference for care location (1); Preferred by patients and families (1); Reduced healthcare costs (1).

Next, physicians were asked to indicate the three most important motivators (or advantages) when considering home-based care (five options were provided). The most common motivator reported was to “reduce unnecessary hospitalizations and observation stays,” with 43 (89.6%) physicians rating this advantage among their top three. The second most common motivator was “reduce risks associated with hospitalization,*”* with 38 (79.2%) rating this in the top three. See Figure 2.

**Figure 3:**
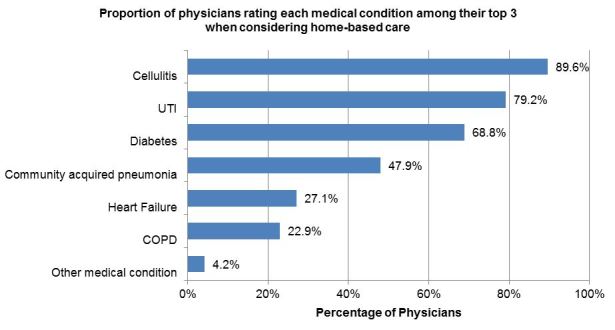
Physician rating of top three medical conditions appropriate for home-based care. Other medical conditions listed: Orthopedic injuries (1); Osteomyelitis (1). *COPD*, chronic obstructive pulmonary disease; *UTI*, urinary tract infection.

Physicians were then asked to indicate the top three medical conditions they would consider for home-based care (six options were provided). The three most common medical conditions reported were cellulitis (43 respondents, 89.6%), *urinary tract* infection (38, 79.2%), and diabetes (33, 68.8%). See Figure 3.

**Figure 4:**
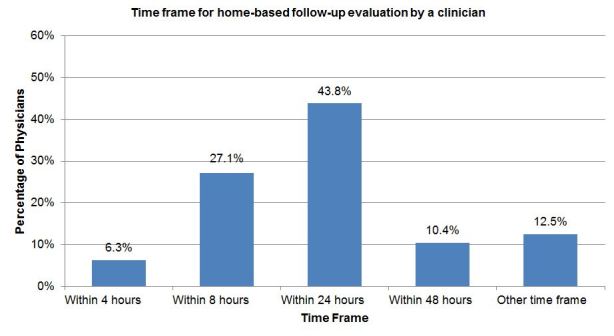
Physicians’ preferred time frame for home-based follow-up clinician evaluation. Other time frames listed: within 12 hours (4); next day (1); case dependent, for example, airway issues (pneumonia/congestive heart failure/chronic obstructive pulmonary disease) likely require earlier care at 8–12hrs, cellulitis 24–48hrs, etc. (1).

The final question asked physicians to indicate how soon a home-based follow-up evaluation by a clinician would need to occur (four options were provided). The most common response indicating the specific time frame within which a physician believed a follow-up evaluation would need to occur was “within 24 hours*”* (21 respondents, 43.8%), followed by “within 8 hours*”* (13, 27.1%). See Figure 4.

### Focus Group Thematic Analysis

Three focus groups were convened with three participants each: (1) with three EPs, (2) with three EP leaders and, (3) with two SWs and one RN. The four individual interviews were conducted with three EPs and one ED RN. The majority of participating EPs had at least some experience in transitioning patients to home health following evaluation in the ED and believed it was a good option to have available. The major themes derived from the focus groups are discussed below and categorized in the table.

### Recognition of a Need for Awareness and Education Regarding Alternatives to Admission

Participants indicated a gap in education of EPs and nursing staff regarding what options exist for home care and the types of services and treatment that can be provided, stating, for example, that “there is a simple lack of awareness [that] we can do it this way” and “awareness is a major factor.” In addition, clinicians wanted to know how to access these services from the ED. One participant stated, “There is no book on case management. There are concepts, there’s a lot of on-the-job training, and it is very situationally different.”

Participants identified the need, due to staff turnover, for ongoing education of new employees and ED residents in regard to home-based care options. Most agreed that to sustain momentum the education also needed to include frequent reminders because “learning goes away after a few months.” The prevailing message was summed up by this physician participant: “In the event of a patient who everyone agrees doesn’t necessarily need to be admitted, but has no good alternative, we, the ED physicians, are also not wanting to admit these people. We would love to have an adequate alternative.”

### Data on the Short- and Longer-Term Benefits of Home-Based Care

Participants felt there were short- and longer-term benefits of home- and community-based care, stating, for example, “Whatever can be addressed at home should be”; “From a social perspective, people do, in my opinion, tend to heal better at home, assuming they have a normal home”; and “There is a realization of so much of what we do in the hospital can really be done at home with appropriate care-takers, and keeping them in the hospital wasn’t going to do a whole lot different than sending them home with some help.” Recognized benefits of home-based care were accompanied by acknowledgment of risk in sending home patients who still need monitoring or interventions. Most physicians felt the patients would be safe as long as they were assured of timely follow-up. “If I could know they’d be seen the next day, I still would [send them home],” and “If I feel that the family and patient are comfortable, I’m not that concerned about medical/legal ramifications.”

### The Need for Streamlined ED Processes to Transition Patients Home

Participants acknowledged the critical need for streamlined processes to transition patients home from the ED. Currently both process and operational barriers exist. In particular, ED operations include time constraints, and participants indicated adding on time-consuming or complicated processes significantly reduces the likelihood the practice would be adopted. ED-to-home transitions must be easy, streamlined, and time-efficient. One physician stated, “A lot of our patients can be taken care of at home, but just due to logistics … sometimes it becomes a challenge.” Another EP said, “Often, we’re really busy down there, and we often take the path of least resistance.” Others expressed standardized order sets, protocols, or algorithms would be helpful.

The strongest recommendation for streamlining was that EDs have someone on site to arrange the transition. The availability of ED case managers—a hard-to-fill position in the hospital due to lack of applicants—was said to be a real problem.

Many explained they’d had difficulty getting assistance even from inpatient case managers. These were several of the comments: “The physical presence of a case manager or social worker in the ED is critical”; “We can avoid the inpatient admission with a series of home health visits. We can actually do this. But without the intermediary of a case manager or social worker involved in the ED, this is very difficult to orchestrate”; “I think we just need a steady case manager presence”; and “Some of them [case managers] have amazing concepts of what can be done at home.”

### The Need for Home-Based Care that is Responsive and Provides Qualified Staff

The need for home-based care to be responsive and provide qualified staff was viewed as extremely important. Some participants discussed challenges in ensuring a qualified home-health workforce. Concern over current compensation structures that may be driving less experienced staff to seek home care jobs was expressed: “Maybe home health doesn’t pay well. Maybe they need to be more comparable to hospitals to have good employees.”

Additional concepts that arose were clinical oversight immediately following the ED encounter, avenues for reimbursement, and regulatory limitations.

Participants also discussed barriers that exist in the broader healthcare ecosystem. Regulations and reimbursement can influence where patients are treated. In order to create new options, participants said, for example, that we “must figure out financial incentives.” Participants articulated that disposition alternatives are limited by what services and treatments get reimbursed and what is easily accessed. Traditional community-based healthcare options (e.g., home health agencies and infusion clinics) typically operate during regular business hours, with little or no responsiveness during the evenings, nights, or weekends. Participants indicated that providing needed care in the most appropriate setting, ED-to-home options need to be available 24 hours a day, 7 days a week. Participants admitted that currently “EDs do not have other good alternatives to admission,” and until those options exist EPs will more often than not default to a hospital admission, even if patients do not require hospital-level care. Participants with experience in ED-to-home transitions found the process “easier if the home health agency is part of the health system.”

The need for a supportive hospital administration was also seen as key to overcoming system barriers because admissions are the main revenue source for hospitals, and efforts to reduce admissions may not be met with support.

In the focus groups, one broad category of possible candidates for home healthcare instead of admission were patients needing intravenous antibiotics for diagnoses such as cellulitis, uncomplicated community-acquired pneumonia, and urinary tract infections. Other diagnoses often mentioned were congestive heart failure and lower extremity deep vein thrombosis. Others indicated that in general the elderly would do better at home whenever possible, and that home care would be a good alternative for patients who were refusing admission and requesting to go home. Most participants agreed that at present the decision to arrange for home care to avoid an admission is made on a case-by-case basis.

## DISCUSSION

Substituting more acute home-based care for hospital-based care has been widely adopted in single-payer systems overseas and to a lesser extent domestically within integrated delivery networks and Veterans Administration hospitals.^10^–^12^ Studies conducted both in the U.S. and abroad show that providing healthcare services in the home is safe, effective, satisfying to patients and clinicians, and lower in cost. 7, 13–16 One such effort is the Hospital at Home® model, which shifts acute care out of the hospital and into the home for select patient cohorts. This model substitutes hospital-level care for a similar level of home-based care.^7^ Broader adoption of this model, especially in non-integrated healthcare systems, has historically been hampered in the U.S. by reimbursement and regulations that have not strongly incentivized short-term, higher-intensity care in the home. Although the body of research on substitutive models demonstrated feasibility, providers seeking hospital-level reimbursement were not successful.^17^

Home-based healthcare, which includes home health, provides skilled nursing and therapy services following an acute hospitalization. It is a widely used lower-cost option compared to both hospitalization and in-home care by a physician. In the U.S., home health is covered as a benefit, provided to both Medicare and Medicaid beneficiaries. The Medicare home health benefit requires both a skilled need and patients meeting home-bound criteria. Although home health services in the U.S. are growing, use of this care delivery option is not widely employed as an alternative for hospital-based care directly following care in the ED. However, the benefit does provide coverage for skilled home-based healthcare services and treatments, including those necessary following ED treatment.

Prior to the present study, little was known regarding the extent to which EPs might recommend home-based delivery of acute medical care. Furthermore, it was not well known whether EPs were aware of what health services are available in the home and whether care at home is a safe and effective option. The present study provides insight into EPs’ knowledge and attitudes toward the option of using home-based healthcare directly from the ED. Nearly three quarters of physicians indicated the main barrier was a lack of processes and help to arrange the transition to home care. Given the number of sick and injured patients coming into the ED, physicians expressed a need for a well-trained support network within the ED, including staff with clear guidelines and infrastructure necessary to transition patients smoothly from ED to the home. EPs expressed a strong motivation to reduce unnecessary admissions and the associated risks of hospitalizations. They also identified the types of patients they consider candidates for home care and the time frame during which they would need the patients to be seen at home.

Focus group participants also identified several barriers that must be overcome to make the ED-to-home option more viable. A knowledge gap exists in that 12 of the physicians either did not know home-based care was an option from the ED, had never encountered the situation, or were not familiar enough with the process to be comfortable using it. Participants acknowledged they encounter patients receiving care in the hospital that could as easily be provided in the home. The main challenges were (1) a lack of time in the ED to arrange home health, (2) a lack of knowledge on how to arrange for home health, and (3) a lack of understanding of the kind of services home health can provide.

In light of recent healthcare outcomes research that has begun to quantify the hazards of hospitalization,^18^–^20^ there is growing belief that certain patients, especially the elderly, might fare better at home. If these higher-risk patients are able to avoid hospitalization, it may reduce their exposure to resistant pathogens, noise, unfamiliar environments, and risk of falls and delirium, and can reasonably be translated into better outcomes and patient satisfaction.

## LIMITATIONS

Several limitations should be considered in the context of this study. First, because the sample size for the survey was small and limited to the relatively narrow setting of an academic medical center, generalizations to nationwide perspectives are beyond the scope of this study. Additionally, individuals selected for interviews that led to development of survey questions were professionals paid for their time to participate. However, these individuals were not informed that their answers would be used to create survey questions.

The survey was intended to uncover thematic areas, first in a local setting, with the potential for surveying a broader sample, and providing the potential for modification and adjustment of the questions. The interview and focus groups were a small convenience sample of self-selected individuals limited to a single institution. These included only three EPs, and therefore may similarly not be representative of the broader population of EPs and ED RNs and SWs. Regional differences in practice patterns, availability of home-based care, and variability in the number and types of personnel staffing EDs may have further biased the responses of the study participants. Surveying a larger, more geographically diverse group of EPs could provide a more balanced perspective.

## CONCLUSION

This study revealed that EPs are generally receptive to referring patients for home-based healthcare following ED treatment to reduce the incidence of “default” hospital admissions. EPs also believe patients with certain diagnoses are likely to benefit and may avoid many of the iatrogenic risks of hospitalization. However, few EPs know specifically what services are available in the home and lack knowledge of the process for invoking an “ED to home-based healthcare” transition. While limited statistical inferences can be drawn from the current survey, the results of this study do serve as a conversation starter, and also form the basis for the design of a larger, nationwide survey. Because the study highlights a general willingness on the part of the EP community, future research should therefore further investigate, on a broader scale, what system-level changes could potentially realize the opportunity for EPs to be provided with a broader range of transition options from the ED. Future research should also explore patient and physician satisfaction with alternative disposition options as well as relevant outcome data (e.g. ED bounce-back rates, need for subsequent hospitalization, etc.). With the emergence of value-based payment reform, more incentives are emerging for both physicians and hospitals to provide these additional options in furtherance of high-value care in the most appropriate setting.

## Figures and Tables

**Table:** Four areas of need identified by focus groups queried about the feasibility of transitioning patients from the emergency department to home-based healthcare.

Knowledge and education regarding:	Efficacy and outcome data for:	Streamlined ED processes	Responsive and qualified home care
Alternatives to admission	Short- and longer-term clinical outcomes	Easy to initiate	Providers can respond within a short time
What home care can provide	Patient and family satisfaction	Efficient ED workflows	Clinicians have skills and expertise to deliver quality care
Accessing home care alternatives	Patient safety	Effective clinician hand-offs	
